# Zinc enhancement of cytidine deaminase activity highlights a potential allosteric role of loop-3 in regulating APOBEC3 enzymes

**DOI:** 10.1038/srep18191

**Published:** 2015-12-18

**Authors:** Ailie Marx, Meytal Galilee, Akram Alian

**Affiliations:** 1 Faculty of Biology, Technion – Israel Institute of Technology, Haifa 320003, Israel

## Abstract

The strong association of APOBEC3 cytidine deaminases with somatic mutations leading to cancers accentuates the importance of their tight intracellular regulation to minimize cellular transformations. We reveal a novel allosteric regulatory mechanism of APOBEC3 enzymes showing that APOBEC3G and APOBEC3A coordination of a secondary zinc ion, reminiscent to ancestral deoxycytidylate deaminases, enhances deamination activity. Zinc binding is pinpointed to loop-3 which whilst highly variable harbors a catalytically essential and spatially conserved asparagine at its N-terminus. We suggest that loop-3 may play a general role in allosterically tuning the activity of zinc-dependent cytidine deaminase family members.

Biology is written in a four-letter nucleotide alphabet that is enriched by a myriad of modifications. One such alteration is the conversion of cytidine to uridine a reaction carried out by the zinc-dependent cytidine deaminase super-family which includes the human apolipoprotein B mRNA editing enzyme catalytic polypeptide-like (APOBEC) proteins. Essential for our complexly evolved life, cellular DNA editing is also increasingly recognized as massively impacting the genetic heterogeneity and chromosomal instability of tumors. Recent high-impact reports show that APOBEC proteins constitute a major cause of somatic mutations leading to various cancers^1^,^2^. The APOBEC family, which deaminates cytidine embedded within a single stranded polynucleotide chain, includes activation-induced cytidine deaminase (AID) acting in the antibody diversification process, APOBEC1 important in lipid metabolism, seven APOBEC3 proteins who play roles in the innate defense against retroviruses, and APOBEC2 and APOBEC4 for whom the physiological substrate and role is yet to be elucidated^3^,^4^,^5^.

APOBEC proteins are not exclusive in their ability to deaminate (de-oxy)nucleotides, forming part of a much wider superfamily of zinc-dependent deaminases including enzymes which convert adenosine to inosine and act on either tRNA (adenosine deaminases acting on tRNA - ADATs) or mRNA (adenosine deaminases acting on RNA - ADARs). In addition to the polynucleotide substrates targeted by the APOBECs, this superfamily also includes cytidine deaminases (CDA’s), which act on free cytidine, and deoxycytidylate deaminases (dCD’s) that deaminate cytidine monophosphate (dCMP), both enzymes being involved in pyrimidine synthesis (reviewed in^3^,^6^).

The conserved catalytic motif and mechanism have both been well detailed: deamination proceeds by a hydrolytic attack on the C4 amine of the substrate by an activating water molecule, which together with three Cys or His residues coordinates a catalytic zinc ion, and a conserved glutamic acid acts as a proton shuttle during catalysis. The conserved core structure of these enzymes includes a backbone of five β-strands and two α-helices, which shape and support the catalytic pocket holding the zinc coordinating histidine and cysteine residues in place. Consequently, the architecture of the substrate-binding cavity is highly preserved with the bound substrates superimposable (reviewed in^3^,^4^,^7^,^8^). The different zinc-dependent cytidine deaminase family members have evolved distinctly around this core to act on different substrates for varying biological roles and under vastly diverse regulations.

Whereas free nucleotide cytidine deaminases have been structurally well characterized both in substrate bound and unbound forms (reviewed in^9^), a structural description of zinc-dependent cytidine deaminases bound to a polynucleotide substrate has remained elusive, despite a growing library of particularly APOBEC proteins. Apart from insights derived from a single TadA-tRNA bound structure revealing a flipped out target base^10^, little is known about the way in which these enzymes contact their polynucleotide substrates, identifying and positioning the target nucleotide for deamination^5^.

Prior to the recent flood of APOBEC structures, structures of deaminases that act on free cytidine were utilized in order to gain insights into the likely conformations of the more evolved APOBEC proteins^3^,^5^. The remarkable structural similarities among the members of this family have long suggested conserved mechanisms by which the substrates, whether in the free form or in the context of polynucleotides, are recognized and deaminated. Although a structural description of a polynucleotide bound APOBEC has remained elusive, it is presumed that the differences in substrate recognition among the family members are mainly a result of the length, composition and position of the loops surrounding the catalytic site: Loop-7 plays an important role in DNA substrate specificity and recognition and loop-1 being widely open in polynucleotide-deaminases allowing for the binding of larger substrates^3^,^4^,^5^,^7^,^8^,^11^,^12^,^13^. The position of loop-3 is especially interesting and apparently plays a critical role in substrate accessibility and binding. NMR resonances of APOBEC3A (A3A) loop-3 residues were found to change upon substrate binding^14^, substituting APOBEC3G (A3G) loop-3 with that from A3A enhanced A3G deamination activity^7^, and a single R256E mutation, which probably disrupts loop-3 conformation, impairs A3G activity^12^. It is notable that loop-3 has been implicated in allosteric control of deamination activity of the ancestral deoxycytidylate deaminases (dCDs)^9^. Although regulation of the APOBEC family of proteins through an allosteric-like mechanism typical to ancient dCDs has not yet been reported, conformational changes and “switches” that could explain the inactivity of individual subunits in an APOBEC3 oligomer have previously been proposed^15^,^16^.

The association of cytidine deaminases with cancer emphasizes the importance of their tight intracellular regulation to minimize the chances of mutations in genomic DNA leading to cellular transformations. Several regulatory mechanisms have been implicated in controlling these enzymes including nuclear cytoplasmic shuttling, regulation of gene expression, recruitment into high molecular mass complexes, interactions with cellular binding partners that can specify targeted DNA loci, and posttranslational modifications including phosphorylation and ubiquitylation, which control their abundance and deaminase activity^1^.

Here we reveal a novel allosteric-like regulation of APOBEC3 deaminases in which the deamination activity is enhanced by the binding of a secondary zinc ion to loop-3. We suggest that the secondary zinc may stabilize loop-3 in a conformation which favorably orients a highly conserved secondary catalytic residue Asn244, located at the N-terminal end of this loop, for substrate binding. Given that loop-3 is the most sequence variable and structurally flexible of the loops surrounding the conserved catalytic core structure of zinc dependent cytidine deaminases, we suggest it may play a role in protein specific regulation by allosteric control of activity.

## Results and Discussion

**Figure 1:**
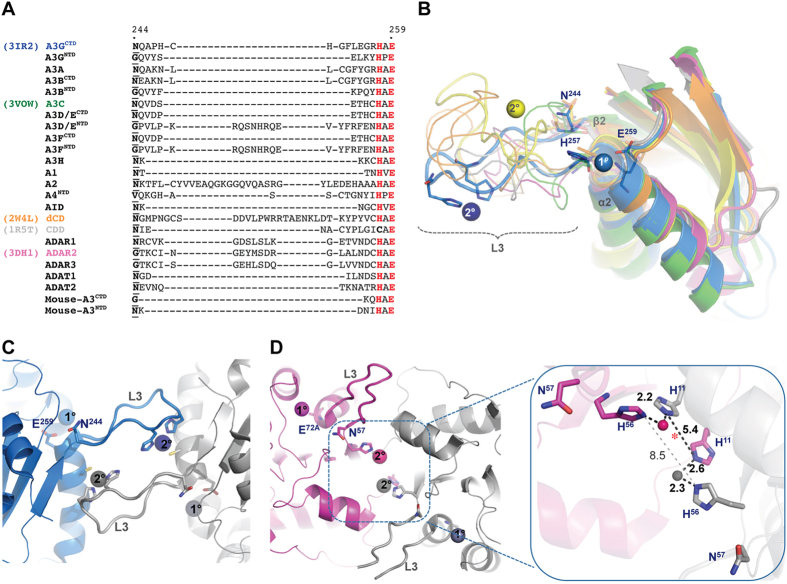
Secondary zinc coordination by the highly variable loop-3. (**A**) Loop-3 sequence alignment of human members of the zinc dependent cytidine deaminase family (and mouse A3). A3G numbering is shown and catalytic H and E residues are highlighted in red. The conserved N (and corresponding residues in inactive enzymes) is in bold font and underlined. (**B**) Structure alignment of representative family members showing conserved catalytic core and loop-3 (L3) with coloring as indicated in (**A**). Conserved N244, H257 and E259 are shown in sticks. Catalytic (1°) and secondary (2°) zinc ions are shown as spheres. (**C**) Dimer interface of A3G^CTD^ (interface-2, 3IR2) mediated by 2° zinc and involving L3. (**D**) Dimer interface of A3A (4XXO) showing secondary 2° zinc mediating the interface and involving L3. Inset: Close up of A3A dimeric interface showing interaction network between N56, H11 and 2° zinc ions. Distances in Å. Red asterisk indicate position of probable zinc ion that might be coordinated by two H11 in H56A mutant.

More than 50% of known human zinc-dependent cytidine deaminase family members have representative structures in the protein data bank (PDB) after recent additions including APOBEC3A (A3A)^14^,^17^, APOBEC3C (A3C)^18^ and APOBEC3F (A3F)^19^,^20^ as well as the long elusive N-terminal domain (NTD) of the two-domain APOBEC3G (A3G)^21^. Alignment of the catalytic core scaffold of these structures highlights loop-3 (A3G C-terminal domain (A3G^CTD^) numbering), which precedes α-2 and is capped at the C-terminus by the (H/C)xE heart of the catalytic motif, as the most variable in length, sequence and conformation (Fig. 1A,B). Remarkably, several crystal structures of the C-terminal domain of A3G^CTD^ (PDB codes: 3IR2, 3V4J, 3V4K) show coordination of a secondary zinc ion via loop-3 analogous to the positioning of a secondary zinc ion in the ancestral dCMP deaminases^16^,^22^ (Fig. 1B). The secondary A3G zinc, which is spatially remote for the catalytic zinc, mediates a dimeric interface (Fig. 1C) and is coordinated by H248 and H250 on loop-3 together with C261 from α-2 of an adjacent molecule. A most recent A3A structure (PDB code: 4XXO) revealed a similar protein-protein organization that places loop-3 of both monomers at the dimer interface, which is also critically mediated by two zinc ions coordinated by H11 and loop-3 H56 (Fig. 1D). While H11A mutation drastically reduced both cooperativity of dimerization and DNA binding affinity (>15 folds), H56A mutation alone lowered only dimerization cooperativity^17^. Structural analysis shows that dimerization cooperativity is mediated by zinc coordination through H56 of one monomer and H11 of another; however, when H56 is mutated dimerization cooperativity may still be facilitated by zinc coordination between H11 residues from each monomer (Fig. 1D inset, asterisk). The two H56 residues are however too far apart (8.5 Å) to support a similar zinc induced dimerization in the absence of H11 (Fig. 1D inset, gray dashed line). Although zinc ions are commonly found in crystal structures as artifacts of the crystallization process and are important in making crystal contacts^23^, it is notable that zinc was not used in the crystallization conditions of these structures although the buffer used in protein sample preparation contained 50 μM zinc acetate^16^,^17^.

### Secondary zinc ion bound to loop-3 enhances deaminase activity of A3G and A3A

**Figure 2:**
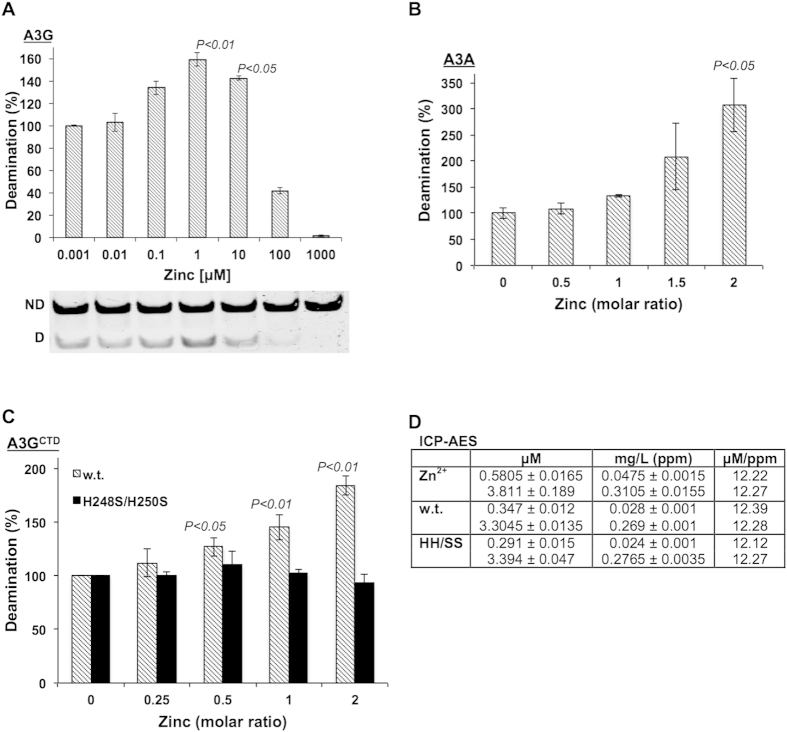
Secondary zinc binding to loop-3 enhances A3G and A3A deaminase activity. Relative deamination activity of full-length A3G (**A**) as a function of increasing zinc concentrations, and of A3A (**B**) at increasing stoichiometric ratios of zinc. A representative gel showing the resolution of non-deaminated “ND” and deaminated “D” substrate of A3G is shown in (**A**). (**C**) Relative deamination activity of A3G^CTD^ w.t. (striped bars) and H248S/H250S mutant (black bars) at increasing stoichiometric ratios of zinc. Data represent mean ± S.E.M. of three biological repeats. P-values (one-way ANOVA) calculated in comparison to control samples (absence of extra zinc). (**D**) ICP-AES data (mg/L) represent means ± S.E.M. of duplicate measurements at two concentrations (μM) of A3G^CTD^ w.t. and H248S/H250S (HH/SS) compared to ZnCl_2_ as a control.

To probe a biologically significance for the secondary zinc observed in the A3 structures we monitored the effect of zinc supplementation on the deamination activity of the full-length two domain A3G and the single domain A3A. Increasing concentrations of zinc distinctively correlate to increased deamination activity in both enzymes (Fig. 2A,B). Although the deamination enhancement effect is clear (160% activity at its peak), the effect of zinc supplementation on A3G is probably understated since the protein has low expression levels in mammalian cells meaning that the final sample was only moderately pure (supplementary zinc may be bound by other protein impurities), therefore, the soluble protein concentration could not be accurately determined (in order to correct for zinc precipitation). In contrast, bacterial overexpression of A3A yielded a highly pure and concentrated protein sample allowing for zinc supplementation at stoichiometric ratios and also for correction of soluble protein concentrations to account for the known precipitating effect of zinc as described in materials and methods. Indeed, this precipitation effect of zinc constrained measurements above the stoichiometric ratios shown in Fig. 2B.

The enhancement of deamination activity by secondary zinc binding was verified and pinpointed to loop-3 by showing that a stoichiometric increase in the concentration of zinc correlated to an increase in the deamination activity of highly pure w.t. A3G^CTD^ but not for the corresponding H248S/H250S mutant (Fig. 2C). Concentrations higher than stoichiometric zinc to protein ratios of 2 resulted in almost total protein precipitation so that deamination activity could not be measured. ICP-AES measurements, on A3G^CTD^ w.t. and H248S/H250S mutant, confirmed that prior to zinc supplementation the primary catalytic zinc is fully occupied and so enhanced deamination activity is not an artifact of reconstituting functional protein (Fig. 2D).

Whereas the crystal structures of secondary bound zinc to A3G^CTD^ and A3A seem to imply that zinc binding induces dimerization, we were unable to detect zinc induced dimerization by homobifunctional (DST and BS^3^, Thermo Scientific Pierce) chemical crosslinking (results not shown). The quaternary structure is noteworthy and significantly different between the different zinc-dependent deaminases where those acting on free bases are generally tetrameric (or in the case of some bacterial dimeric) and those acting on dCMP are hexameric^24^,^25^. A3′s reside in different oligomeric states and although the monomeric form is suggested as the most active there is no clear consensus as to their functional oligomerization state^4^,^17^,^26^. Conceivably, oligomerization plays an important role in modifying the position and flexibility of the loops surrounding the active site and so too may play an important role in regulation of deamination activity. Based on zinc titration coupled with NMR analysis, it was suggested that zinc might mediate the equilibrium of oligomeric and monomeric states in A3G^CTD^^16^. A different study using atomic force microscopy probed the role of ssDNA in A3G oligomerization and found that a H248A/H250A double mutant displayed a similar pattern of oligomerization to w.t. protein however with reduced DNA binding stability^27^. Most recently, it was found that cooperative dimerization of A3A regulates specific binding of ssDNA and this binding is an order of magnitude higher when a substrate cytidine is present^17^. The significantly higher affinity for the substrate than the product could explain the difficulty in capturing dimerization in equilibrium experiments.

The novel finding that a secondary zinc ion can stimulate A3 deamination activity provides an important insight into the specific mechanics of A3G and A3A but more importantly this result suggests a family wide potential for loop-3 driven allosteric control of cytidine deamination that might have biological implications with regard to controlling the activity of these proteins in normal and diseased cells.

### Loop-3 plays a role in correctly orienting the secondary catalytic Asn for deamination activity

 Contrasting the overall sequence and conformational variability of loop-3, is the presence of an asparagine residue (Asn244 in A3G), located at the N-terminus end of loop-3, which is completely spatially conserved in the reportedly active Zn-domains across the entire family (Fig. 1A)^13^. Although Asn244 has been recognized as essential for activity with alanine substitution abolishing activity in A3G, A3A and AID^12^,^28^,^29^, it has only recently been suggested as a secondary catalytic residue in APOBEC3 proteins^13^.

**Figure 3:**
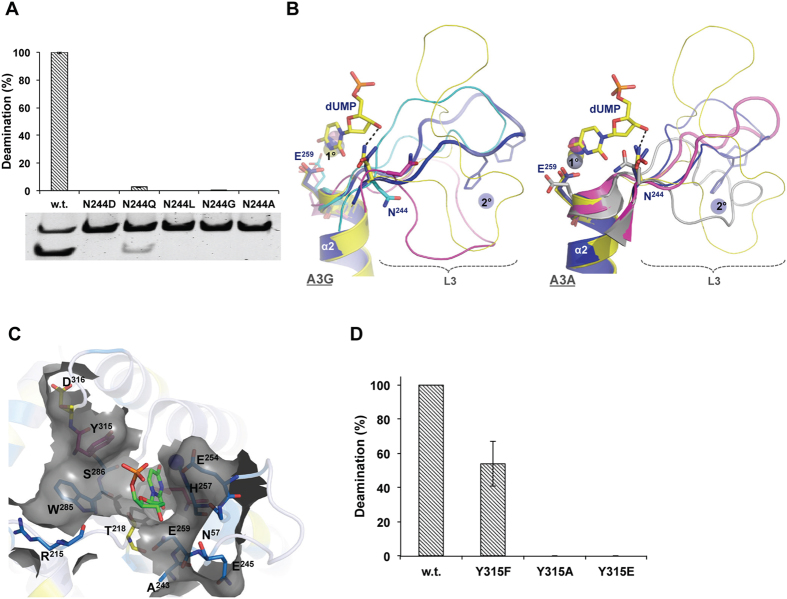
Asn244 is essential for A3G^CTD^ activity. (**A**) Relative deamination activity of A3G^CTD^ w.t. and N244 mutants. Error bars represent 3 biological repeats and a representative gel resolving non-deaminated “ND” and deaminated “D” substrate is shown. (**B**) Structural alignment of A3G^CTD^ (left) and A3A (right). A3G^CTD^ bound (blue: 3IR2) or unbound (magenta: 2JYW, cyan: 2KEM) to a 2° Zn. Shown in yellow is a dCMP deaminase (4P9C) bound to dUMP ligand. A3A monomeric (gray: 2M65) and dimeric (magenta: 4XXO) structures are compared. Shown in blue and yellow are the A3G^CTD^ and dCMP deaminase, respectively. The conserved N244 and E259 are shown in sticks. Catalytic (1°) and 2° zinc ions are shown as spheres. Black dashed line depicts hydrogen bonding between N43 and the dUMP ligand. (**C**) A3G residues most affected by zinc titration (blue/yellow residues in^16^) are presented in sticks and gray-surface. dUMP in sticks to depict substrate binding site. Y315 and catalytic E259 in magenta sticks. (**D**) Relative deamination activity of A3G^CTD^ w.t. and Y315 mutants. Error bars represent 3 biological repeats.

To clarify the essentiality of the nitrogen moiety and the role of the side chain length of this Asn in crucially optimizing activity we created various A3G^CTD^ N244 mutants and assessed the effect on deamination activity. Mutation of Asn244 to Ala, Gly, Leu or Asp abolished activity whereas mutation to Gln retained 3% of w.t. activity (Fig. 3A). These results corroborate with the suggestion that the carbonyl group of deoxycytidine (dC) is imperatively stabilized by hydrogen bonding with the amide group of Asn^13^, an interaction observed in the substrate bound free nucleotide structures^9^ as well as the tRNA bound TadA structures^10^. Asn mutation to the similarly sized Asp abolished activity with the loss of the amide group however the longer amide containing Gln retained some minimal activity (Fig. 3A) suggesting that whilst the presence of the amide group is fundamental the side chain length is highly critical.

The importance of Asn amide moiety and side chain length is not surprising and is also reflected by its very precise and conserved positioning in substrate bound structures^9^. In apo structures this Asn occupies variable orientations. A3G^CTD^ and A3A crystal structures demonstrate that where a secondary zinc ion is bound, this Asn takes on the substrate bound conformation (Fig. 3B). We hypothesize that loop-3 conformation may favorably orient this Asn, and other residues surrounding the catalytic site, to properly dock the bound substrate with precise coulomb distances priming the protein for productive catalysis. The variability of A3 loops with regards to flexibility, length and composition has been suggested to affect the catalytic site by modulating the positions and conformations of residues lining these catalytic pockets^13^. The most recent A3G^CTD^ crystal structures (PDB codes: 4ROV and 4ROW), documenting a novel head-to-tail orientation^8^, revealed H248/H250 of loop-3 mediating, albeit without zinc, an interface of weak hydrophobic interactions with P210/W211 on loop-1 of the next monomer and further stressed the importance of loop-3 in stabilizing the catalytic core. Mutating P210 to either Gly or Ala, in order to disrupt this interaction, almost ablated catalytic activity. Similarly, increasing loop-3 flexibility by D264A (which eliminates D264/R256 salt bridge), F252A (which stabilizes R256 by hydrophobic interaction), Q245A (adjacent to N244 at the N-terminal tip of loop-3) and R256A (adjacent to H257 of catalytic core at the C-terminal tip of loop-3) mutations destabilized the catalytic center and reduced or abolished deaminase activity^8^. One possibility is that zinc binding may stabilize loop-3 conformation favorable for this positioning and so enhancing activity. Interestingly, enzymes that utilize cofactor proteins can apparently maintain functional stability and some minimal levels of activity in Asn absence. For example, while substituting Asn with Ala in rabbit APOBEC1, which employs ACF cofactor, abolishes activity on ssDNA, it only reduces activity by 80% on the biological substrate apoB 100 mRNA^30^. Likewise, ADAR2 does not harbor Asn244 but is active only in the presence of the cofactor IP_3_^31^.

In addition to Asn244, NMR analysis of zinc titration extends the zinc effect to residues predominantly residing on loop-3, α-2 and residues surrounding the catalytic pocket^16^ (Fig. 3C). These residues include H257 (coordinates the catalytic zinc), R215, W285, S286, and T218, which have all previously been determined as crucial for deamination activity^8^,^13^,^32^,^33^. Recently, the corresponding residues in AID were dubbed “secondary catalytic residues” since, while not participating directly in the deamination reaction, they apparently have a critical role in docking and stabilizing the substrate in the catalytic groove^13^. The necessity for precise substrate docking can also explain how deamination is inhibited in A3G when T218 (and AID T27) is phosphorylated or mutated to Glu without affecting DNA binding^33^. A longer side chain at this position would clash with the substrate preventing proper alignment necessary for deamination or preventing the presumable base flipping of the target dC into the catalytic site. Similarly, the two aromatic residues W285 (AID W84) and Y315 (AID Y114) are crucial for base stacking the substrate dC inside the catalytic pocket^13^. Although Y315 did not show NMR chemical shift perpetuations upon zinc titration^16^, Y315 has been shown crucial for deamination with a Y315A mutation in A3G eliminating activity^12^. The analogous substitution in A3A not only eliminates deaminase activity but also inhibits DNA binding^34^. A3DE has a Cys residue at this position and its substitution by Tyr increases anti viral activity more than 20 fold^35^. The role of Y315 in stabilizing the substrate appears to involve both essential base stacking and preferable hydrogen/electrostatic interactions. We found that retaining only the charge at this position (Y315E) abolishes deamination activity while removing the charge and retaining the aromatic ring (Y315F) reduces activity to ~60% (Fig. 3D).

Efficient deamination relies on the precise orientation of functional residues whose position may be dictated by the conformation of loop-3. Stabilizing this loop and reducing fluctuations apparently increases activity. Presumably, an interaction which similarly holds loop-3 in an unfavorable orientation would hinder deamination activity.

### A G56N mutation at the N-terminus end of loop-3 in the inactive NTD of A3G does not bestow deamination activity

**Figure 4:**
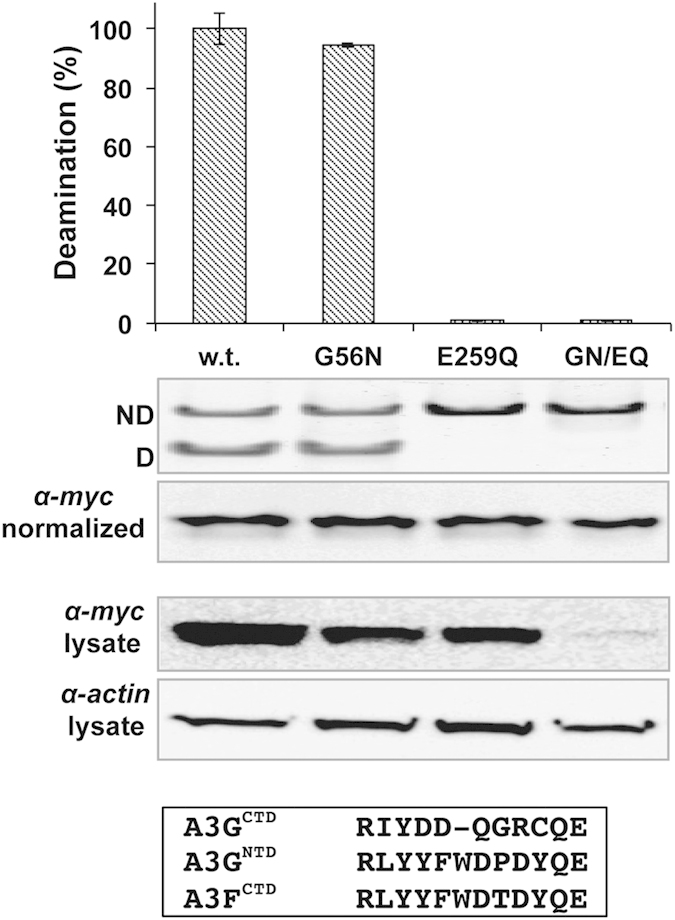
A G56N mutation to the NTD of full-length A3G does not bestow deamination activity. Relative deamination activity of full-length A3G w.t. and mutants: G56N, E259Q and the double mutant G56N/E259Q (GN/EQ). Error bars (S.E.M) represent three biological repeats. Protein concentrations were normalized prior to assessing deamination activity and standardization is demonstrated by α-myc of purified samples (α-myc normalized). α-myc on the lysate demonstrates significantly decreased expression of A3G G56N/E259Q mutant on a comparable α-actin background. Sequence alignment of loop-7 from A3G^CTD^, A3G^NTD^ and A3F^CTD^ is shown.

Inactive domains of zinc-dependent cytidine deaminases, which otherwise contain the catalytic motif, generally harbor Gly in place of Asn244 (Fig. 1A). Given the essentiality of Asn for activity and that N244G abolished activity in A3G^CTD^ (Fig. 3A) we investigated if the reverse mutation can restore activity in the NTD of the inactive A3G E259Q mutant. The G56N substitution failed to restore deamination activity to the inactive full-length A3G E259Q on the preferred CC substrate (Fig. 4). Additionally, since A3G^NTD^ loop-7, dictating substrate specificity, has a similar sequence to A3F (Fig. 4) who favor deamination of a TC motif, we assessed deamination on either TC or the AID preferred TWC substrates, neither of which was deaminated by the A3G G56N/E259Q double mutant. It is noteworthy that the double mutant was repeatedly expressed at much lower levels than either of the corresponding single mutants or the w.t. protein and required the use of many more cells to obtain comparable quantities of protein to carry out deamination reactions (Fig. 4, α-myc lysate).

Apparently, A3G^NTD^ harbors additional as yet un-clarified constraints that hinder deamination activity. The recent NMR structure of solubilized A3G^NTD^ (PBD code: 2MZZ)^21^ suggests that lack of activity results from aberrations in α-2, mainly the positioning of the catalytic Glu and a tilting towards the C-terminus end of α-3^21^. However, given that this structure was obtained by mutating 20% of the w.t. residues, care must be taken in analyzing fine structural depictions. Alternatively, it has been suggested that the volume of the catalytic cavity positively corresponds with activity (A3G^NTD^ having the smallest pocket)^36^. Indeed, mere accessibility to the catalytic pocket has been implicated as crucial in dictating A3/AID deaminase activity^13^.

Although steric hindrance caused by the relative orientations of the NTD and CTD cannot be entirely precluded, such an explanation is weakened by the fact that in mice it is the NTD of A3 that is active^37^. A dedicated study using domain swapping variants of full-length A3G will be required to address this possibility.

In conclusion, these findings demonstrates that loop-3 has the potential to allosterically regulate the cytidine deaminase activity of A3′s, a feature generally attributed as unique to dCMP deaminases. Here, allosteric enhancement of deamination activity was demonstrated *in vitro* for A3G and A3A by the binding of a secondary zinc ion to loop-3. We predict however that *in vivo* different ions, cofactors or proteins may similarly interact with A3A, A3G and other members of the A3 family in order to enhance or even decrease cytidine deamination activity. Zinc-dependent cytidine deaminases are diverse in their roles and must carry out the conserved chemical reaction at the right time and place with aberrations to these dedicated functions already shown to have disastrous pathogenic outcomes^2^. It will be interesting to probe the role of loop-3 in future studies aimed at exploring regulation of zinc-dependent cytidine demainases and particularly if an allosteric-like regulatory A3 feature might be exploited by cellular pathways and infectious pathogens. For example, could allosteric control of loop-3 provide an explanation to a reported non-degradative pathway inhibition of APOBEC3/AID by Vif 38,39?

## Methods

### Sequence and structure alignment

Loop-3 sequences were identified manually from the structures (or homology models) of each enzyme and were subsequently aligned using T-Coffee^40^. Structure alignment and illustrations were created in PyMOL Molecular Graphics System (Schrödinger, LLC).

### Protein expression and purification

Wild type (w.t.) and mutant variants of A3G^CTD^ (residues 191–384) and w.t. A3A were subcloned into pET28b (Novagen) and expressed in ArcticExpress cells (Agilent Technologies Genomics) with 0.5 mM IPTG induction overnight at 16 °C (supplemented with 50 μM zinc acetate). The His-tagged A3A and A3G^CTD^ were purified using HisPur Cobalt Resin (Thermo Fisher Scientific) in a buffer containing 500 mM NaCl, 50 mM Potassium Phosphate pH 7.5 and 5 mM β-mercaptoethanol and dialyzed into a final buffer containing 150 mM NaCl, 20 mM Tris-HCl pH 7.0 and 1 mM tris(2-carboxyethyl)phosphine (TCEP). A3G^CTD^ was further purified by size exclusion chromatography.

Full-length A3G was subcloned into pcDNA3.1 (Thermo Fisher Scientific Invitrogen) and expressed with a C-terminal Myc/His tag in human embryonic kidney (HEK) 293 T cells, harvested 48 hours post transfection and purified as previously described^26^. Cells were broken by 3 cycles of freeze-thaw in lysis buffer (50 mM Tris, pH 8.0, 1 mM PMSF, 10% (v/v) glycerol and 0.8% (v/v) NP-40) and soluble fraction was adjusted to 0.8 M NaCl and treated with 50 μg/ml RNaseA (30 min at 37 °C) prior to purification using Ni-NTA (Thermo Fisher Scientific). Elution buffer contained 50 mM Tris, pH 8.0, 0.3 M NaCl, 10% (v/v) glycerol, 250 mM imidazole and subsequently dialyzed into a final buffer containing 50 mM Tris pH 8, 0.3 M NaCl, 1 mM DTT.

### Cytidine deamination assay

Cytidine deamination activity on ssDNA was assessed using the established PCR-based assay with a protein concentration adjusted to an overall product formation of ~10% activity to ensure assay sensitivity as described before^26^. Deamination reactions (37 °C) were performed in a total volume of 10 μl in 25 mM Tris pH 7.0, 1ul protein, 0.1 μg/μl BSA and 1 pmol/μl ssDNA substrate containing CCC, TTC or TGC deamination motifs. The deamination reaction mixture (1ul) was amplified by PCR, incubated (1 h at 37 °C) with the StuI, DraI or XbaI restriction enzymes (New England Biolabs) respectively to cleave the deaminated product, which was resolved from the undeaminated (uncleaved) substrate on a polyacrylamide gel.

### Zinc supplementation to deamination assays

Zinc acetate was incubated with purified proteins in stoichiometric ratios, centrifuged and subsequent deamination activity was calculated after correction for soluble protein concentration to account for the known precipitating effect of zinc^41^. However, because of the less purity of full-length A3G produced from mammalian cells, which hampered accurate determination of protein concentration, it was not possible to consider stoichiometric ratios (as in Fig. 2B,C), but rather molar concentrations (Fig. 2A), of supplemented zinc acetate. Full occupancy of catalytic Zn^2+^ was measured by ICP-AES (ICP Spectrometer, iCap 6000 Series, Thermo Scientific) on affinity-purified samples, which had been dialysed twice against a buffer containing 20 mM Hepes pH 7.0, 150 mM NaCl and 1 mM EDTA to remove non-specifically bound metal ions. Duplicate ICP-AES measurements conducted using two different concentrations (~0.48 and ~3.8 μM) of w.t. and H248S/H250S mutant against corresponding zinc acetate concentrations as standards.

## Additional Information

**How to cite this article**: Marx, A. *et al.* Zinc enhancement of cytidine deaminase activity highlights a potential allosteric role of loop-3 in regulating APOBEC3 enzymes. *Sci. Rep.* **5**, 18191; doi: 10.1038/srep18191 (2015).
